# Cardio-Metabolic Indices and Metabolic Syndrome as Predictors of Clinical Severity of Gastroenteropancreatic Neuroendocrine Tumors

**DOI:** 10.3389/fendo.2021.649496

**Published:** 2021-03-18

**Authors:** Luigi Barrea, Giovanna Muscogiuri, Roberta Modica, Barbara Altieri, Gabriella Pugliese, Roberto Minotta, Antongiulio Faggiano, Annamaria Colao, Silvia Savastano

**Affiliations:** ^1^Dipartimento di Scienze Umanistiche, Università Telematica Pegaso, Napoli, Italy; ^2^Centro Italiano per la cura e il Benessere del paziente con Obesità (C.I.B.O), Endocrinology Unit, Department of Clinical Medicine and Surgery, University Medical School of Naples, Naples, Italy; ^3^Unit of Endocrinology, Dipartimento di Medicina Clinica e Chirurgia, Federico II University Medical School of Naples, Naples, Italy; ^4^Division of Endocrinology and Diabetes, Department of Internal Medicine I, University Hospital, University of Würzburg, Würzburg, Germany; ^5^Department of Clinical and Molecular Medicine, Sapienza University of Rome, Rome, Italy; ^6^Cattedra Unesco “Educazione alla salute e allo svilupposostenibile“, University Federico II, Naples, Italy

**Keywords:** gastroenteropancreatic neuroendocrine tumors, visceral adiposity index, fatty liver index, cardio-metabolic indices, metabolic syndrome

## Abstract

**Background:**

Obesity, mainly visceral obesity, and metabolic syndrome (MetS) are major risk factors for the development of type 2 diabetes, cardiovascular diseases, and cancer. Data analyzing the association of obesity and MetS with gastroenteropancreatic neuroendocrine neoplasms (GEP-NEN) are lacking. Fatty liver index (FLI) is a non-invasive tool for identifying individuals with non-alcoholic fatty liver disease (NAFLD). Visceral adiposity index (VAI) has been suggested as a gender-specific indicator of adipose dysfunction. Both indexes have been proposed as early predictors of MetS. This study aimed to investigate the association of FLI VAI as early predictors of MetS with gastroenteropancreatic neuroendocrine tumors (GEP-NETs).

**Methods:**

A cross-sectional, case–control, observational study was carried out at the ENETS Centers of Excellence Multidisciplinary Group for Neuroendocrine Tumors, University “Federico II”. VAI and FLI were calculated.

**Results:**

We enrolled 109 patients with histologically confirmed G1/G2 GEP-NET (53 M; 57.06 ± 15.96 years), as well as 109 healthy subjects, age, sex- and body mass index-matched. Forty-four GEP-NET patients were G2, of which 21 were with progressive disease, and 27 patients had metastases. GEP-NET patients had a higher value of VAI (*p* < 0.001) and FLI (*p* = 0.049) and higher MetS presence (*p* < 0.001) compared with controls. VAI and FLI values and MetS presence were higher in G2 than in G1 patients (*p* < 0.001), in patients with progressive disease, and in metastatic *vs* non-metastatic patients (*p* < 0.001). In addition, higher values of VAI and FLI and higher MetS presence were significantly correlated with the worst clinical severity of NENs. The cut-off values for the FLI and MetS to predict high grading of GEP-NETs and the presence of metastasis were also provided.

**Conclusions:**

This is the first study investigating an association between VAI and FLI as early predictors of MetS and GEP-NET. Our findings report that the worsening of clinicopathological characteristics in GEP-NET is associated with higher presence of MetS, NAFLD, evaluated by FLI, and visceral adiposity dysfunction, evaluated by VAI. Addressing the clinical evaluation of MetS presence, NAFLD, and visceral adiposity dysfunction might be of crucial relevance to establish targeted preventive and treatment interventions of NEN-related metabolic comorbidities.

## Introduction

Neuroendocrine neoplasms (NENs) represent a group of tumors characterized by a wide biological variability and clinical heterogeneity. NENs originate from the cells of the neuroendocrine system and they can arise in all tissues and organs; however, the gastroenteropancreatic (GEP) and respiratory tracts are the most frequently affected sites (1). Of all malignant cancers, NENs represent only 2%, although recent epidemiological data report a progressive increase in their incidence (2). When NENs are not associated with any endocrine syndrome, their diagnosis may be delayed for years by non-specificity of presenting, with the frequent progression to a metastatic stage prior to clinical diagnosis (3–5).

Very recently, the classification of the World Health Organization (WHO) recognized three forms of well-differentiated GEP-NENs, classified as G1, G2, and G3 neuroendocrine tumors (NETs), based on the proliferative activity expressed by the Ki67 index (6). GEP-NETs have a variable aggressiveness and are associated with a good to moderate survival, but poorly differentiated NENs, the so-called neuroendocrine carcinoma (NEC), are associated with a higher Ki67 index and a poorer prognosis (6).

The association of environmental factors, including obesity, mainly visceral obesity, metabolic syndrome (MetS), and non-alcoholic fatty liver disease (NAFLD), the hepatic manifestation of MetS (7), has been implicated as risk factors for different cancers (8, 9). However, the amount of evidence concerning their possible role as risk factors in NEN pathogenesis is still limited. There is an expanding interest towards the effect of diets on body composition, metabolic parameters, and oxidative status (10). Evidence suggests that there are multiple sources of oxidative stress in obesity, and it may have an influence on carcinogenesis (11). Polyphenols counteract oxidative stress, and the potential protective effect of substances as resveratrol has been investigated (12, 13). The link between obesity and cancer involves possible epigenetic modulators (14, 15), and it could impact on novel therapeutic approaches.

Growing results show the relationship between MetS or its components with several types of cancer development and cancer-related mortality (16, 17). It is suggested that the adipokines secreted from visceral adipocyte dysfunction, and the development of NAFLD play a key role in this association (18).

The gold standard for the diagnosis of NAFLD is the liver biopsy, even though non-invasive techniques, such as magnetic resonance imaging, computed tomography, and liver ultrasonography report adequate concordance with histological results (19). The fatty liver index (FLI), a simple algorithm based on parameters that are routine measurements in clinical practice, such as body mass index (BMI), waist circumference (WC), triglycerides (TGs) and glutamyltransferase (GGT), shows a high concordance with the liver imaging techniques and the histological criteria representing a useful tool to predict the presence of NAFLD (20). In addition, since most variables included in this algorithm are also traditional risk factors for cardiovascular diseases (CVD), FLI has also proved to be an early marker of CVD (21).

Visceral fat and liver inflammation are strictly associated in patients with NAFLD (22). Visceral Adiposity Index (VAI) is considered a marker of adipose tissue dysfunction based on BMI, WC in association with functional parameters such as TG and high-density lipoprotein cholesterol (HDL) (23–25). Similarly to FLI, VAI is associated with MetS (26) and several metabolic diseases, including type 2 diabetes mellitus (27). Of interest, both FLI and VAI have been used in several studies as predictors of incident cancer (28–31). In particular, in a very recent study, high FLI values have been reported to predict NAFLD and breast cancer risk in postmenopausal women (29), while in a population-based longitudinal study VAI was reported as a predictor of incident colorectal cancer (32).

One of the relevant and as yet poorly investigated aspects of the pathogenesis of GEP-NEN is the possible involvement of metabolic dysfunctions, including NAFLD and MetS, particularly in GEP-NETs (G1 and G2), which have a natural history very different from NEC (33). Very recently, Santos AP et al. reported the highest presence of MetS and single risk factors, including WC, fasting plasma glucose, and fasting TG in 96 patients with GEP-NET compared with a control group cross-matched for age and gender (34). However, there is no evidence to date that has evaluated FLI and VAI in patients with NEN, either on their role as an early predictors of MetS.

Based on these premises, this case–control, cross-sectional study aims to investigate the alteration of cardio-metabolic indices, such as VAI and FLI, as early markers for the diagnosis of visceral adiposity dysfunction and NAFLD, respectively, in patients with GEP-NET. In addition, we investigated the possible association of VAI, FLI, and MetS on the clinical severity of NET. Finally, we provided specific cut-offs for cardiometabolic indices to predict grading, presence of metastases, and disease status.

## Materials and Methods

### Design and Setting

This cross-sectional case–control observational study was carried out at the Department of Clinical Medicine and Surgery, Unit of Endocrinology, European Neuroendocrine Tumor Society (ENETS) Center of Excellence Multidisciplinary Group for Neuroendocrine Tumors, University “Federico II” of Naples. Both GEP-NET patients and controls were recruited from May 2017 to January 2020. The “Federico II” Medical School Ethical Committee has approved this cross-sectional case–control observational study (n. 201/17), which was conducted in accordance with the Code of Ethics of the World Medical Association (Declaration of Helsinki) for experiments involving humans. The purpose of the study was explained to all participants, and a written informed consent was obtained.

### Population Study

The study has been conducted on 218 adult Caucasian subjects, in particular 109 GEP-NET patients and 109 healthy individuals as a control group enrolled among hospital volunteers and in the opera prevention project (35), and employees from the same geographical area.

The control group were matched by demographic and anthropometric characteristics, including sex, age, and BMI. In addition, none of the participants had a history of cancer, liver or renal failure, chronic inflammatory diseases, alcohol abuse, and none of them assumed medicaments. In addition, none of the individuals of the control group contemporarily participated in other clinical trials during the period of this study to avoid overlapping enrollment.

To improve the power and homogeneity of this study, only patients meeting the following criteria were included:

- Histological diagnosis of well-differentiated, low grade (G)1 and G2 GEP-NET, according to the classification of by the WHO (6);
- Patients with functioning GEP-NET: biochemically free of disease, without medical treatment, or after surgery performed more than 6 months before recruitment;
- Patients with non-functioning GEP-NET: at the moment of diagnosis treatment-naïve or after endoscopic surgery performed more than 6 months before the recruitment, or discontinuing Somatostatin Analogs (SSAs) for more than 6 months.

Instead were excluded GEP-NET patients meeting one or more of the following criteria:

- Well-differentiated/high grade G3 GEP-NET or poorly differentiated neuroendocrine carcinomas at histological diagnosis, according to WHO classification (6), since it has been reported that grade G3 GEP-NET patients were at risk of malnutrition (36);
- Diagnosis of Merkel cell carcinoma, pheochromocytoma/paraganglioma, medullary thyroid cancer, bronchial, or thymic NET;
- Ongoing medical treatment at the moment of the visit, including SSAs or targeted therapy, since it has been reported that these therapies could affect motor and absorptive functions, gastrointestinal secretory, or cause anorexia and hepatic toxicity (5);
- Individuals who underwent major surgery, since it could change the anatomy of the gastrointestinal tract;
- Patients with functioning GEP-NET who have not undergone gastrointestinal curative surgery for less than 6 months before recruitment and that were not pharmacologically treated at the moment of recruitment with drugs that affect the secretion of hormones (peptides and amines) which could cause dysfunction of the gastrointestinal tract, including altered motility, diarrhea, steatorrhea, and malabsorption (5);
- Based on a complete medical examination and laboratory investigation, the presence of clinical diseases that could influence metabolism, including liver or renal failure, acute or chronic inflammatory diseases, and history of other types of cancer;
- Abuse of alcohol intake defined by the DSM-V criteria (37).

### Power Size Justification

The power of the sample was calculated by the difference of means ± standard deviation (SD) of the number of risk factors of MetS between GEP-NET and control group (2.06 ± 1.52 *vs* 0.97 ± 1.13; respectively). Considering that the number of cases required in GEP-NET and control group was 102, we have set at 109 the number of patients for GEP-NET and at 109 individuals for the control group.

The calculated power size was 95%, with a type I (alpha) error of 0.05 (95%), and a type II (beta) of 0.05. The calculations of sample size and power were performed while using a sample size calculator Clinical Calc (38), as previously reported (39–42).

### Physical Activity and Smoking Habits

Physical activity were evaluated by a standard questionnaire that expressed whether the participant habitually engaged at least 30 min/day of aerobic exercise (YES/NO), as already reported in several other previous studies (43–45). Similarly, through a standard questionnaire, individuals were considered as ‘former smokers’ when they stopped smoking at least one year before the interview, ‘current smokers’ when smoking at least one cigarette per day, and ‘non-current smokers’, as previously reported (46–48). Former and non-current smokers were considered as ‘no-smokers’ for the analyses.

### Anthropometric Measurements

Anthropometric measurements were obtained with participants wearing light clothes and without shoes. Height and body weight were measured to the nearest 1 cm using a wall-mounted stadiometer and derived to the nearest 50 g using a calibrated balance beam scale, respectively (Seca 711; Seca, Hamburg, Germany). BMI was calculated by weight and height [weight (kg) divided by height squared (m^2^), kg/m^2^]. According to WHO’s criteria, participants were classified by BMI as normal weight (BMI 18.5–24.9 kg/m^2^), overweight (BMI 25.0–29.9 kg/m^2^), grade I obesity (BMI 30.0–34.9 kg/m^2^), grade II obesity (BMI 35.0–39.9 kg/m^2^), grade III obesity (BMI ≥ 40.0 kg/m^2^), as previously reported (49–52).

In line with the National Center for Health Statistics (NCHS), WC was measured to the closest 0.1 cm at the natural indentation or at a midway level between the lower edge of the rib cage and the iliac crest if no natural indentation was visible using a non-stretchable measuring tape (53).

### Blood Pressure and Criteria to Define MetS

Systolic (SBP) and Diastolic (DBP) Blood Pressures were measured in all participants three times, and the mean of the second and third reading was recorded after the subject had been sitting for at least 10 min, with a random sphygmomanometer (Gelman Hawksley Ltd., Sussex, UK), as explained in other previous studies (54–56).

MetS was diagnosed according to the National Cholesterol Education Program Adult Treatment Panel (NCEP ATP) III definition if three or more of the following five criteria are present: WC ≥102 cm (men) or 88 cm (women), blood pressure ≥130/85 mmHg, fasting TG level ≥150 mg/dl, fasting HDL cholesterol level ≤40 mg/dl (men) or ≤50 mg/dl (women), and fasting glucose ≥100 mg/dl (57).

### Cardio-Metabolic Indices

VAI score has been calculated by the following sex-specific formula. Both triglycerides and HDL levels were expressed in mmol/L. Age-specific VAI cut-off values were used according to Amato MC et al. (23, 58).

*Males: VAI* = [*WC*/39.68 + (1.88 * *BMI*)] * (*TG*/1.03) * (1.31/*HDL*)

*Females: VAI* = [*WC*/36.58 + (1.89*BMI)] * (*TG*/0.81) * (1.52/*HDL*)

FLI was calculated with the formula: [FLI = eL/(1 + eL) × 100, L = 0.953 × loge TG + 0.139 BMI + 0.718 × loge*γ*GT + 0.053 × WC-15.745]. FLI of 30 was considered as the cut-off value on the basis of Bedogni’s criterion (59).

### Assay Methods

After an overnight fast of at least 8 h, samples were collected in the morning between 8 and 10 a.m. and stored at −80°C until being processed. All biochemical analyses were performed with a Roche Modular Analytics System in the Central Biochemistry Laboratory of our Institution. Low-Density Lipoprotein (LDL) cholesterol and HDL cholesterol were determined by a direct method (homogeneous enzymatic assay for the direct quantitative determination of LDL and HDL cholesterol).

### Clinicopathological Characteristics of the Tumor

In this study, we enrolled patients with G1–G2 GEP-NET, collecting data about primary tumor site, mitotic count, Ki67 index, tumor size, stage, genetic syndromes as multiple endocrine neoplasia type 1 (MEN1), presence of metastases or clinical functioning syndromes, comorbidities, and therapies for each patients.

Tumor size (mm) was calculated as the maximum diameter in the pathological specimen of the tumor or in the last imaging (computed tomography or magnetic resonance imaging) when surgery was not performed. In patients with multiple pancreatic lesions, as in MEN1, we considered the diameter of the biggest nodule. Only in a few cases (n = 3) the tumor size was not defined since the primary lesion was not found.

Tumor grade followed WHO 2010 classification, and tumor stage was defined according to the ENETS criteria, and patients were classified with localized disease (stages I–III) or advanced disease (presence of metastases, stage IV) (60). Immunohistochemistry for chromogranin A, synaptophysin, and Ki67 index was performed in all formalin-fixed paraffin-embedded tissue samples from biopsy or surgery of the primary tumor and/or metastases (61).

At the time of the evaluation, patients were classified as ‘disease-free’ when there was no biochemical or morphological evidence of disease, ‘stable disease’ or ‘progressive disease’ according to RECIST 1.1 criteria (62).

### Statistical Analysis

The data distribution was evaluated by Kolmogorov–Smirnov test and the abnormal data (age, BMI, Ki67 index, SBP, DBP, fasting glucose, HDL-cholesterol, VAI, FLI, and MetS) were normalized by logarithm. The abnormal variables were logarithmically transformed and back-transformed for presentation in tables and figures.

The chi-square (χ^2^) test was used to determine the significance of differences in the frequency distribution in gender, smoking, physical activity, difference in cardio-metabolic indices and MetS between patients and controls, difference in cardio-metabolic indices and MetS among G1 and G2, NET patients free of the disease or with stable disease, and presence or absence of metastasis.

Student’s paired *t*-test was used to analyze differences among age, anthropometric measurements, blood pressure, metabolic profile, cardio-metabolic indices, and MetS between GEP-NET patients and control group, for the difference among parameters included in this study with tumor grading (G1 *vs* G2), and presence/absence of metastasis, followed by Bonferroni *post hoc* analysis.

The differences among age, anthropometric measurements, blood pressure, metabolic profile, cardio-metabolic indices, and MetS with disease status (progressive disease, free disease, and stable disease) were analyzed by ANOVA test followed by the Bonferroni *post-hoc* test.

Proportional Odds Ratio (OR) models, *p*-value, 95% Interval Confidence (IC), and R^2^ were performed to assess the association among quantitative variables (G1 *vs* G2 and presence/absence of metastasis). A multinomial logistic regression analysis, χ^2^, *p*-value, and Akaike Information Criterion (AIC), and R^2^ was performed to model the association among age, anthropometric measurements, blood pressure, metabolic profile, cardio-metabolic indices and MetS with the three groups of disease status (disease free, stable disease and progressive disease).

In addition, three multiple linear regression analysis models (stepwise method), expressed as R^2^, beta (*β*), and *t*, with tumor grading, metastasis, and disease status as dependent variables were used to estimate the predictive value of VAI, FLI, and MetS.

Receiver operator characteristic (ROC) curve analysis was performed to determine the sensitivity and specificity, criterion, standard error, and area under the curve (AUC), as well as cut-off values for MetS and FLI in detecting tumor grading (G2) and presence of metastasis in the GEP-NET patients. Variables with a variance inflation factor (VIF) >10 were excluded to avoid multicollinearity. Values ≤5% were considered statistically significant. Data were analyzed using the MedCalc^®^ package (Version 12.3.0 1993- 2012, MedCalc Software bvba–MedCalc Software, Mariakerke, Belgium) and SPSS Software (PASW Version 21.0, SPSS Inc., Chicago, IL, USA).

## Results

### Demographic, Clinical Characteristics, and Metabolic Parameters of GEP-NET Patients and Control Group

**Figure 1:**
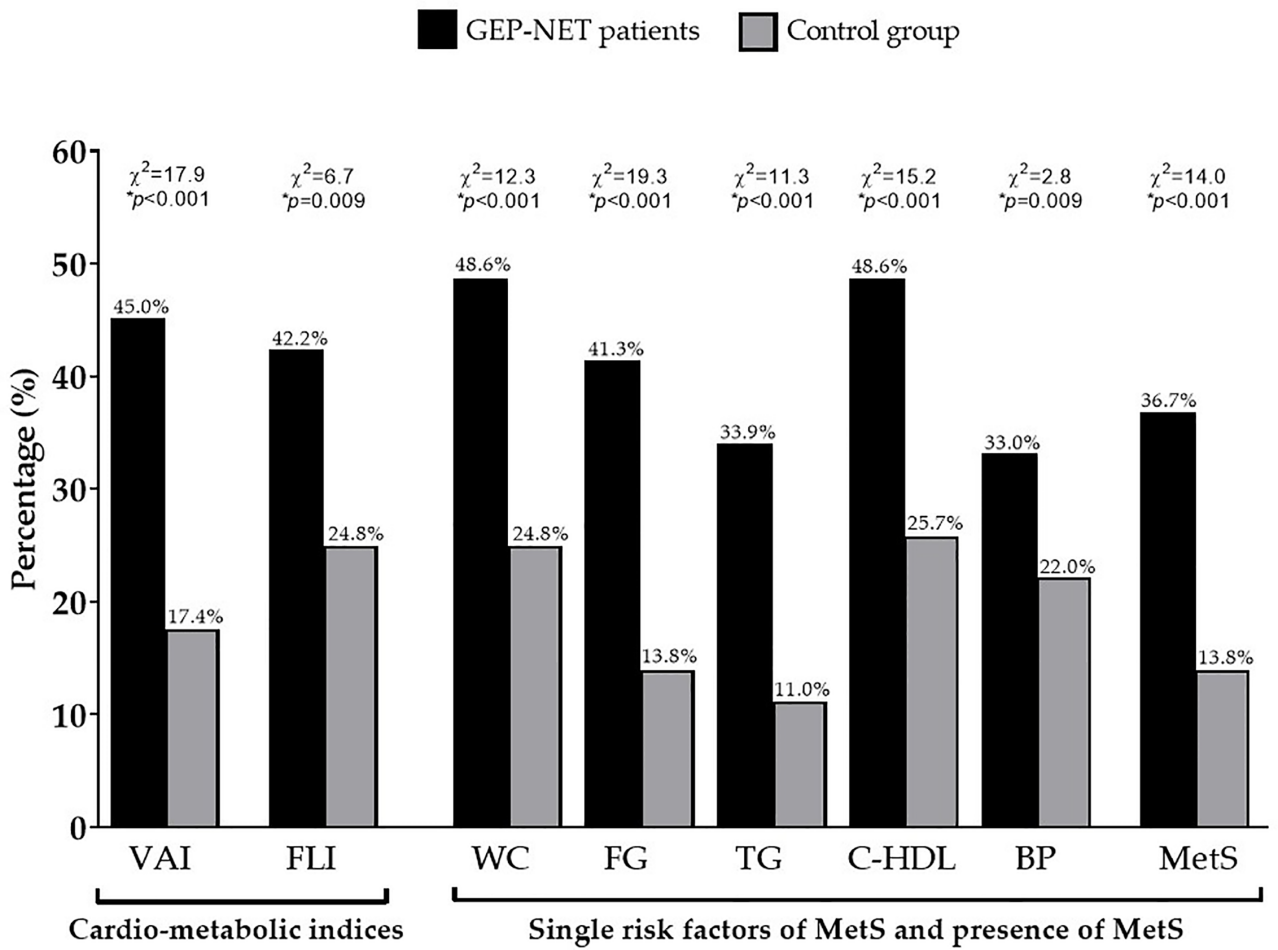
Percentage differences in cardiometabolic indices, single risk factors of MetS, and presence of MetS in GEP-NET patients compared to controls. Most percentage of GEP-NET patients presented visceral adipose dysfunction (*p* < 0.001). Similarly, the percentage of presence of NAFLD in GEP-NET patients was higher than in the control group (*p* = 0.009). In addition, both single risk factors of MetS and the presence of MetS (*p* < 0.001) were more frequently diagnosed among GEP-NET patients than in controls. GEP-NET, Gastroenteropancreatic Neoplasm; VAI, Visceral Adiposity Index; FLI, Fatty Liver Index; WC, Waist Circumference; FC, Fasting glucose; TG, Triglycerides; C-HDL, Cholesterol-High Density Lipoprotein; BP, Blood Pressure; MetS, Metabolic Syndrome.

Demographic, clinical characteristics, and metabolic parameters of GEP-NET patients compared to controls were shown in **Table 1**. Of note, GEP-NET patients presented significant differences in comparison to the control group, in particular smoked less (*p* < 0.001), presented higher WC (*p* = 0.004) and SBP (*p* = 0.007), a worse metabolic profile, and had higher cardio-metabolic indices and MetS (**Table 1**). **Figure 1** shows the percentage differences in cardiometabolic indices, single risk factors of MetS, and presence of MetS in GEP-NET patients compared to controls. Considering age-and-gender specific cut-off points of VAI, most percentage of GEP-NET patients presented visceral adipose dysfunction (*p* < 0.001). Similarly, the percentage of presence of NAFLD in GEP-NET patients was higher than in the control group (*p* = 0.009). In addition, both single risk factors of MetS and the presence of MetS were more frequently diagnosed among GEP-NET patients than in controls (*p*=0.001), as reported in **Figure 1**.

**Table 1:** Demographic, clinical characteristics, and metabolic parameters of GEP-NET patients compared to the control group.

Parameters	GEP-NET patients n. 109	Control Group n. 109	*p*-value
**Demographic characteristics**			
Gender (Females)	56 (51.4%)	56 (51.4%)	χ^2^ = 0.018, *p* = 0.892
Age (Years)	57.06 ± 15.96	56.16 ± 12.89	0.370
**Clinical characteristics**			
Smoking (Yes)	37 (33.9%)	67 (61.5%)	χ^2^ = 15.46, ***p* < 0.001**
Physical activity (Yes)	49 (45.0%)	54 (49.5%)	χ^2^ = 0.29, *p* = 0.587
**Anthropometric measurements**			
BMI (kg/m^2^)	27.55 ± 5.33	28.15 ± 4.07	0.364
WC (cm)	93.87 ± 14.74	88.38 ± 10.93	**0.004**
**Blood pressure**			
SBP (mmHg)	125.18 ± 11.96	120.50 ± 12.41	**0.007**
DBP (mmHg)	76.74 ± 7.71	75.50 ± 7.93	0.209
**Metabolic profile**			
Fasting Glucose (mg/dl)	108.13 ± 15.49	92.35 ± 14.58	**<0.001**
Total cholesterol (mg/dl)	190.86 ± 41.78	158.97 ± 30.86	**<0.001**
HDL cholesterol (mg/dl)	46.75 ± 15.29	50.29 ± 8.05	**0.034**
LDL cholesterol (mg/dl)	118.70 ± 40.02	86.74 ± 31.22	**<0.001**
Triglycerides (mg/dl)	127.07 ± 51.55	109.70 ± 28.87	**0.003**
**Cardio-Metabolic indices and MetS**			
VAI	2.29 ± 1.57	1.53 ± 0.70	**<0.001**
FLI	52.15 ± 29.52	44.36 ± 23.32	**0.049**
MetS (number parameter)	2.06 ± 1.52	0.97 ± 1.13	**<0.001**

GEP-NET, Gastroenteropancreatic Neoplasm; BMI, Body Mass Index; WC, Waist Circumference; SBP, Systolic Blood Pressure; DBP, Diastolic Blood Pressure; HDL, High-Density Lipoprotein; LDL, Low-Density Lipoprotein; VAI, Visceral Adiposity Index; FLI, Fatty Liver Index; MetS, Metabolic Syndrome. A p value in bold type denotes a significant difference (p < 0.05).

### Tumor Characteristics of GEP-NET Patients

A total of 109 patients (F:M = 56:53) affected by GEP-NET were included in the study. The mean size of the tumor was 24.58 ± 22.71 mm. Primary NETs were located in the pancreas (n = 54, 49.5%), stomach (n = 17, 15.6%), intestine (n = 30, 27.6%), and in few cases the primary site was unknown (n = 8, 7.3%). The majority of patients had non-functioning GEP-NET (n = 97, 89.0%). Twenty-two NET patients (20.2%) had a MEN1 syndrome. All GEP-NET patients were classified according to the pathological parameters with the mitotic rate and Ki67 index, as well differentiated tumor G1 (n = 65, 59.6%) or G2 (n = 44, 40.4%); the mean of Ki67 index was 3.88 ± 4.08%. At diagnosis, 27 patients (24.8%) had metastases (stage IV), the majority of them in the liver. At the time when the patients were enrolled in the clinical study, most of them (n. 51, 46.8%) had stable disease, 37 patients (33.9%) were disease free, and the remaining 21 patients (19.3%) had progressive disease, according to the RECIST1.1 criteria.

### Cardio-Metabolic Indices and MetS in GEP-NET Patients According to Tumor Grading, Presence of Metastasis, and Disease Status

Differences in demographic, anthropometric measurements, blood pressure, metabolic parameters, and cardio-metabolic indices and MetS in the GEP-NET patients grouped by grading G1/G2 were summarized in **Table 2**.

**Table 2:** Differences in demographic, anthropometric measurements, blood pressure, metabolic parameters, and cardio-metabolic indices and MetS in the GEP-NET patients according to tumor grading.

Parameters	G1 n. 65	G2 n.44	*p*-value
**Age (years)**	55.32 ± 17.26	59.64 ± 13.61	0.149
**Anthropometric measurements**			
BMI (kg/m^2^)	27.23 ± 5.64	28.02 ± 4.88	0.439
WC (cm)	90.58 ± 15.15	98.73 ± 12.77	**0.003**
**Blood pressure**			
SBP (mmHg)	121.85 ± 10.52	130.11 ± 12.37	**<0.001**
DBP (mmHg)	75.08 ± 7.47	79.20 ± 7.47	**0.006**
**Metabolic profile**			
Fasting Glucose (mg/dl)	102.71 ± 14.22	116.14 ± 13.84	**<0.001**
Total cholesterol (mg/dl)	178.52 ± 32.34	209.09 ± 47.51	**<0.001**
HDL cholesterol (mg/dl)	48.97 ± 13.21	43.48 ± 17.59	0.066
LDL cholesterol (mg/dl)	105.97 ± 29.27	137.49 ± 46.28	**<0.001**
Triglycerides (mg/dl)	117.91 ± 43.98	140.61 ± 59.01	**0.023**
**Cardio-Metabolic indices and MetS**			
VAI	1.89 ± 1.05	2.88 ± 1.99	**0.001**
FLI	42.93 ± 28.15	65.77 ± 26.27	**<0.001**
MetS (number parameter)	1.42 ± 1.12	3.00 ± 1.56	**<0.001**

GEP-NET, Gastroenteropancreatic Neoplasm; G, grading; BMI, Body Mass Index; WC, Waist Circumference; SBP, Systolic Blood Pressure; DBP, Diastolic Blood Pressure; HDL, High-Density Lipoprotein; LDL, Low-Density Lipoprotein; VAI, Visceral Adiposity Index; FLI, Fatty Liver Index; MetS, Metabolic Syndrome. A p value in bold type denotes a significant difference (p < 0.05).

**Figure 2:**
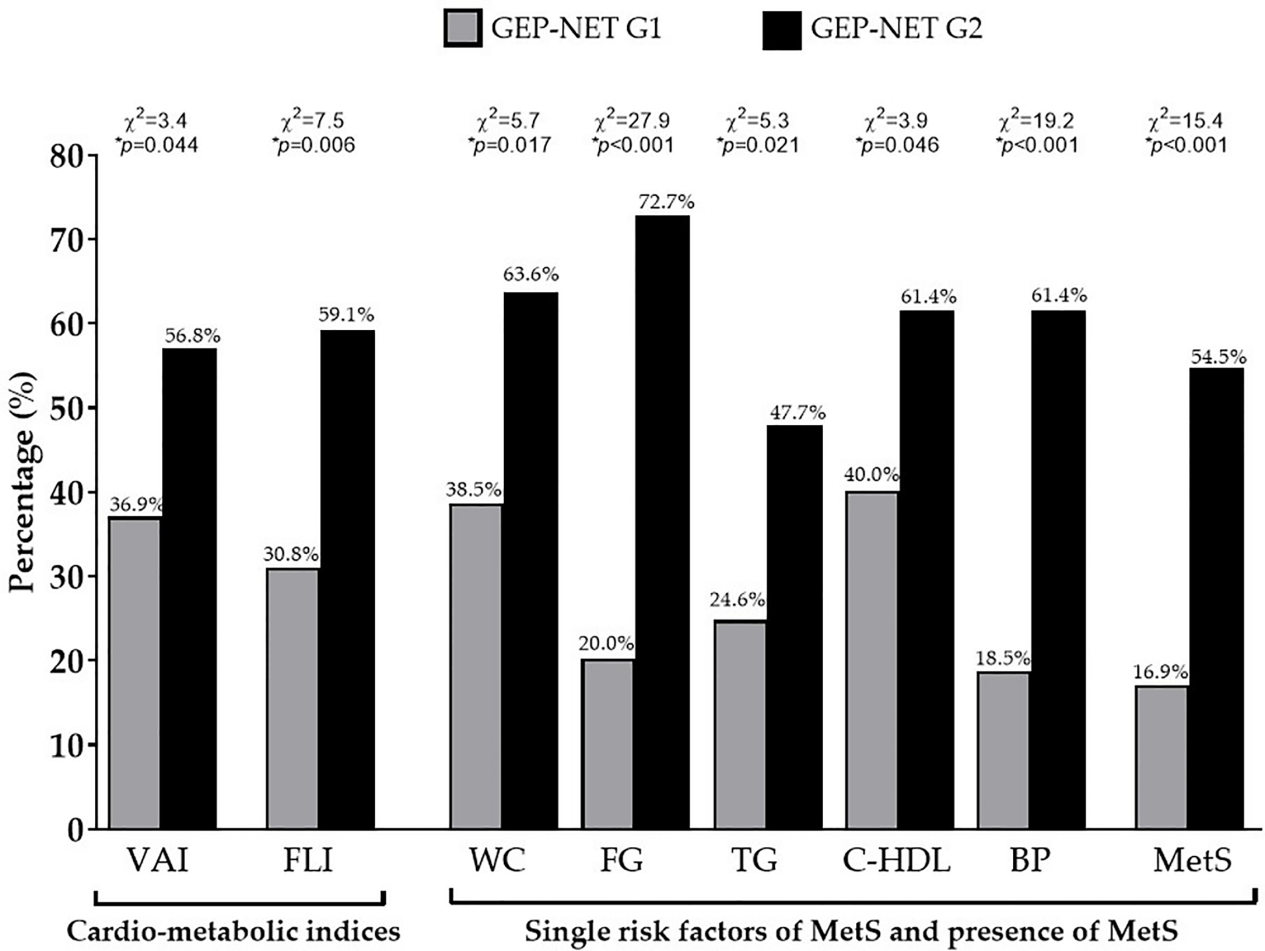
Difference of VAI, FLI, and MetS according to the grading. GEP-NET G2 patients presented the highest percentage of cardio-metabolic indices (*p* = 0.044 and *p* = 0.006 for VAI and FLI, respectively), single risk factors of MetS, and presence of MetS (*p* < 0.001) compared to GEP-NET G1 patients. GEP-NET, Gastroenteropancreatic Neoplasm; G, Grading, VAI, Visceral Adiposity Index; FLI, Fatty Liver Index; WC, Waist Circumference; FC, Fasting glucose; TG, Triglycerides; C-HDL, Cholesterol-High Density Lipoprotein; BP, Blood Pressure; MetS, Metabolic Syndrome.

Interestingly, GEP-NET G2 patients in comparison to patients with localized GEP-NET G1, had significant higher WC (*p* = 0.003), SBP and DBP (*p* < 0.001 and *p* = 0.006, respectively), and the worst metabolic parameters, except HDL cholesterol. Of interest, GEP-NET G2 patients showed the highest value of cardio-metabolic indices and MetS (number parameter) (**Table 2**). Similarly, **Figure 2** reported the difference of VAI, FLI, and MetS according to specific cut-off points. As observed, GEP-NET G2 patients presented the highest percentage of cardio-metabolic indices, single risk factors of MetS, and presence of MetS (*p* < 0.001) compared to GEP-NET G1 patients.

Similar data were observed also when these parameters were grouped by disease status (**Table 3**). A significant worse metabolic profile, cardio-metabolic indices, and MetS were shown in GEP-NET patients with progressive disease, in comparison to patients who were free of disease or with stable disease (**Table 3**).

**Table 3:** Differences in demographic, anthropometric measurements, blood pressure, metabolic parameters, and cardio-metabolic indices and MetS in the GEP-NET patients according to disease status.

Parameters	Progressive Disease n. 21 (19.3%)	Free Disease n. 37 (33.9%)	Stable Disease n. 51 (46.8%)	*p*-value
**Age (years)**	56.95 ± 13.51	55.05 ± 17.37	58.57 ± 15.95	0.598
**Anthropometric measurements**				
BMI (kg/m^2^)	29.07 ± 4.81	27.26 ± 4.83	27.13 ± 5.85	0.353
WC (cm)	98.64 ± 13.75	92.53 ± 15.62	92.88 ± 14.35	0.256
**Blood pressure**				
SBP (mmHg)	127.62 ± 12.61	122.70 ± 12.22	125.98 ± 11.40	0.263
DBP (mmHg)	78.33 ± 8.99	75.41 ± 7.85	77.06 ± 7.01	0.354
**Metabolic profile**				
Fasting Glucose (mg/dl)	119.81 ± 17.42	104.65 ± 12.93	105.84 ± 14.31	**<0.001**
Total cholesterol (mg/dl)	215.05 ± 42.99	193.86 ± 41.00	178.73 ± 37.66	**0.003**
HDL cholesterol (mg/dl)	41.81 ± 16.35	42.27 ± 13.46	52.04 ± 14.64	**0.004**
LDL cholesterol (mg/dl)	142.70 ± 44.65	124.32 ± 37.98	104.74 ± 34.06	**<0.001**
Triglycerides (mg/dl)	152.71 ± 62.74	136.38 ± 43.69	109.76 ± 46.32	**0.002**
**Cardio-Metabolic indices and MetS**				
VAI	3.12 ± 2.02	2.69 ± 1.67	1.66 ± 0.93	**<0.001**
FLI	69.24 ± 31.58	51.97 ± 28.13	45.24 ± 27.22	**0.006**
MetS (number parameter)	3.19 ± 1.78	2.11 ± 1.34	1.55 ± 1.27	**<0.001**

GEP-NET, Gastroenteropancreatic Neoplasm; G, grading; BMI, Body Mass Index; WC, Waist Circumference; SBP, Systolic Blood Pressure; DBP, Diastolic Blood Pressure; HDL, High-Density Lipoprotein; LDL, Low-Density Lipoprotein; VAI, Visceral Adiposity Index; FLI, Fatty Liver Index; MetS, Metabolic Syndrome. A p value in bold type denotes a significant difference (p < 0.05).

**Figure 3:**
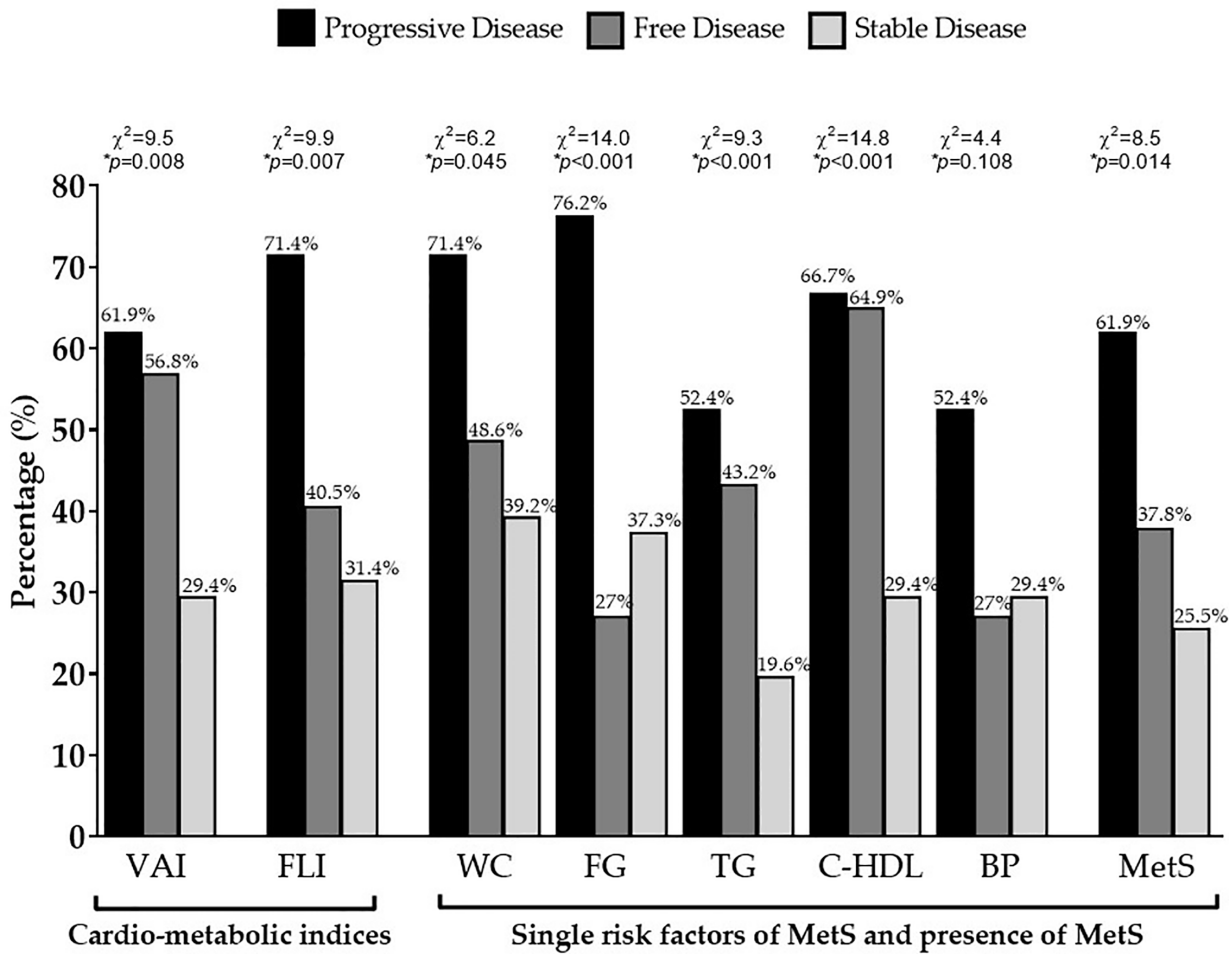
Difference of VAI, FLI, and MetS according to status of disease. GEP-NET patients with progressive disease had the highest percentage of cardio-metabolic indices (*p* = 0.008 and *p* = 0.007 for VAI and FLI, respectively), single risk factors of MetS, and presence of MetS (*p*=0.014) compared to GEP-NET patients free of disease or with stable disease. VAI, Visceral Adiposity Index; FLI, Fatty Liver Index; WC, Waist Circumference; FC, Fasting glucose; TG, Triglycerides; C-HDL, Cholesterol-High Density Lipoprotein; BP, Blood Pressure; MetS, Metabolic Syndrome.

Even when we considered the difference of VAI, FLI, and MetS according to specific cut-off points, GEP-NET patients with progressive disease had the highest percentage of cardio-metabolic indices, single risk factors of MetS and presence of MetS (*p* = 0.014) compared to GEP-NET patients free of the disease or with stable disease, **Figure 3**.

**Figure 4:**
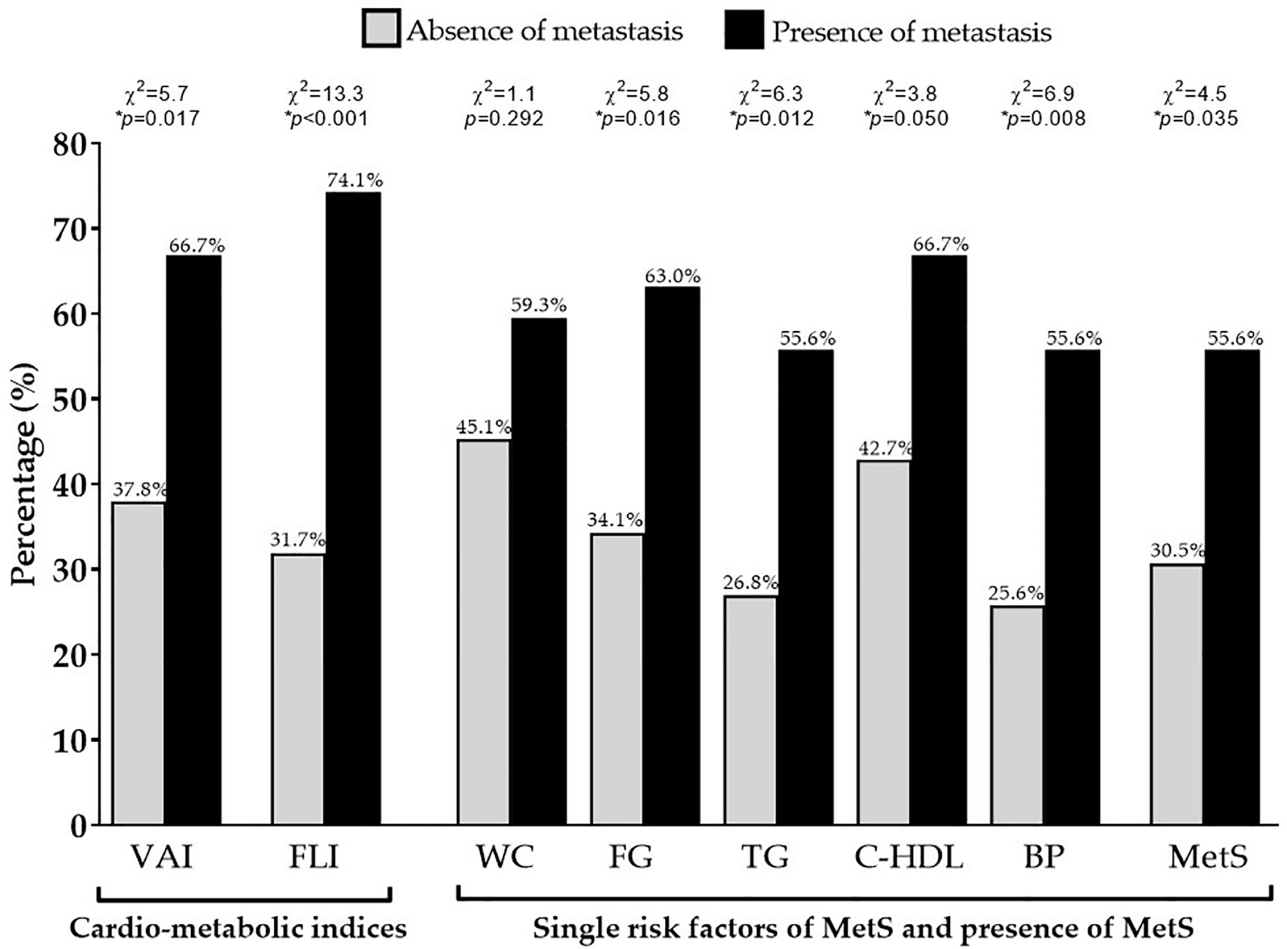
Difference of VAI, FLI, and MetS according to metastasis. GEP-NET patients with the presence of metastasis had the highest percentage of cardio-metabolic indices (*p* = 0.017 and *p* < 0.001 for VAI and FLI, respectively), single risk factors of MetS (except for the WC, *p* = 0.292), and presence of MetS (*p* = 0.035) compared to GEP-NET patients with the absence of metastasis. VAI, Visceral Adiposity Index; FLI, Fatty Liver Index; WC, Waist Circumference; FC, Fasting glucose; TG, Triglycerides; C-HDL, Cholesterol-High Density Lipoprotein; BP, Blood Pressure; MetS, Metabolic Syndrome.

Differences in demographic, anthropometric measurements, blood pressure, metabolic parameters, and cardio-metabolic indices and MetS in the GEP-NET patients according to the presence/absence of metastasis were summarized in **Table 4**. The worse WC (*p* = 0.003), blood pressure, metabolic profile, cardio-metabolic indices, and MetS were presented in the presence of metastasis, and the latter GEP-NET patients also presented the highest percentage of cardio-metabolic indices, single risk factors of MetS, and presence of MetS (*p* = 0.035), **Figure 4**.

**Table 4:** Differences in demographic, anthropometric measurements, blood pressure, metabolic parameters, and cardio-metabolic indices and MetS in the GEP-NET patients according to the presence/absence of metastasis.

Parameters	Absence of Metastasis n. 82	Presence of Metastasis n. 27	*p*-value
**Age (years)**	56.48 ± 16.86	58.85 ± 12.94	0.448
**Anthropometric measurements**			
BMI (kg/m^2^)	27.17 ± 5.44	28.70 ± 4.93	0.182
WC (cm)	92.16 ± 14.63	99.08 ± 14.05	**0.033**
**Blood pressure**			
SBP (mmHg)	123.59 ± 11.66	130.00 ± 11.76	**0.018**
DBP (mmHg)	75.73 ± 6.94	79.81 ± 9.14	**0.016**
**Metabolic profile**			
Fasting Glucose (mg/dl)	105.43 ± 15.12	116.29 ± 13.87	**0.001**
Total cholesterol (mg/dl)	184.64 ± 36.84	209.74 ± 50.29	**0.006**
HDL cholesterol (mg/dl)	48.59 ± 14.73	41.14 ± 15.88	**0.027**
LDL cholesterol (mg/dl)	112.09 ± 34.20	138.75 ± 49.53	**0.002**
Triglycerides (mg/dl)	119.78 ± 46.84	149.22 ± 59.39	**0.009**
**Cardio-Metabolic indices and MetS**			
VAI	2.00 ± 1.20	3.14 ± 2.17	**0.001**
FLI	46.59 ± 28.42	69.04 ± 26.62	**<0.001**
MetS (number parameter)	1.74 ± 1.35	3.00 ± 1.64	**<0.001**

GEP-NET, Gastroenteropancreatic Neoplasm; G, grading; BMI, Body Mass Index; WC, Waist Circumference; SBP, Systolic Blood Pressure; DBP, Diastolic Blood Pressure; HDL, High-Density Lipoprotein; LDL, Low-Density Lipoprotein; VAI, Visceral Adiposity Index; FLI, Fatty Liver Index; MetS, Metabolic Syndrome. A p value in bold type denotes a significant difference (p < 0.05).

### Correlation Between Tumor Aggressiveness and Metabolic Profile, Cardio-Metabolic Indices, and MetS in GEP-NET Patients

To assess the correlation of grading and metastasis, a bivariate proportional OR model with demographic, anthropometric measurements, blood pressure, metabolic profile, cardio-metabolic indices, and MetS was performed (**Table 5**). A part age, BMI, and HDL cholesterol for grading all other parameters were significantly associated with the highest grading G2 and with the presence of metastasis; **Table 5**.

**Table 5:** Bivariate proportional odds ratio model performed to assess the association of tumor aggressiveness with demographic, anthropometric measurements, blood pressure, metabolic profile, cardio-metabolic indices, and MetS.

Parameters	Grading G2				Metastasis (presence)			
	OR	*p*-value	95% CI	R^2^	OR	*p*-value	95% CI	R^2^
**Age (years)**	1.02	0.168	0.993–1.04	0.02	1.01	0.501	0.98–1.04	0.01
**Anthropometric measurements**								
BMI (kg/m^2^)	1.03	0.250	0.957–1.11	0.01	1.05	0.206	0.97–1.14	0.02
WC (cm)	1.04	**0.006**	1.01–1.07	0.07	1.03	**0.038**	1.00–1.07	0.04
**Blood pressure**								
SBP (mmHg)	1.08	**0.001**	1.03–1.11	0.12	1.05	**0.018**	1.00–1.09	0.05
DBP (mmHg)	1.07	**0.007**	1.02–1.14	0.07	1.08	**0.019**	1.01–1.14	0.05
**Metabolic profile**								
Fasting Glucose (mg/dl)	1.07	**<0.001**	1.04–1.11	0.19	1.05	**0.003**	1.02–1.08	0.09
Total cholesterol (mg/dl)	1.02	**<0.001**	1.01–1.03	0.13	1.02	**0.008**	1.00–1.03	0.07
HDL cholesterol (mg/dl)	0.98	0.070	0.95–1.00	0.03	0.96	**0.032**	0.93–0.99	0.05
LDL cholesterol (mg/dl)	1.02	**<0.001**	1.01–1.03	0.15	1.02	**0.004**	1.00–1.03	0.08
Triglycerides (mg/dl)	1.00	**0.026**	1.00–1.02	0.05	1.01	**0.012**	1.00–1.02	0.06
**Cardio-Metabolic indices and MetS**								
VAI	1.56	**0.003**	1.17–2.09	0.10	1.56	**0.003**	1.16–2.09	0.09
FLI	1.03	**<0.001**	1.01–1.05	0.14	1.03	**0.001**	1.01–1.05	1.11
MetS (number parameter)	2.30	**<0.001**	1.63–3.23	0.25	1.77	**<0.001**	1.29–2.43	0.12

BMI, Body Mass Index; WC, Waist Circumference; SBP, Systolic Blood Pressure; DBP, Diastolic Blood Pressure; HDL, High-Density Lipoprotein; LDL, Low-Density Lipoprotein; VAI, Visceral Adiposity Index; FLI, Fatty Liver Index; MetS, Metabolic Syndrome. A p value in bold type denotes a significant difference (p < 0.05).

A multinomial logistic regression model to assess the association between patients with progressive disease and demographics, anthropometric measurements, blood pressure, metabolic profile, cardio-metabolic indices, and MetS, was performed (**Table 6**). Progressive disease was associated with higher values of age (*p* = 0.012), WC (*p* = 0.005), blood pressure (*p* = 0.007 and *p* = 0.004 for SBP and DBP, respectively), fasting glucose (*p* = 0.015), triglycerides (*p* = 0.029), VAI (*p* = 0.001), FLI (*p* = 0.009), MetS (*p* < 0.001), and lower HDL cholesterol (*p* = 0.001); **Table 6**.

**Table 6:** Multinomial logistic regression model to assess the association between disease status with age, anthropometric measurements, blood pressure, metabolic profile, cardio-metabolic indices, and MetS.

Parameters	Progressive disease			
	χ^2^	*p* value	AIC	R^2^
Age (years)	134.68	**0.012**	178.05	0.709
**Anthropometric measurements**				
BMI (kg/m^2^)	239.50	0.094	239.49	0.889
WC (cm)	189.59	**0.005**	212.11	0.824
**Blood pressure**				
SBD (mmHg)	38.85	**0.007**	79.07	0.300
DBD (mmHg)	32.18	**0.004**	65.46	0.256
**Metabolic profile**				
Fasting Glucose (mg/dl)	132.94	**0.015**	176.48	0.705
Total cholesterol (mg/dl)	183.17	0.172	208.65	0.814
HDL cholesterol (mg/dl)	138.19	**0.001**	176.07	0.719
LDL cholesterol (mg/dl)	222.86	0.109	231.18	0.871
Triglycerides (mg/dl)	208.99	**0.029**	224.25	0.853
**Cardio-Metabolic indices and MetS**				
VAI	122.86	**0.001**	131.18	0.771
FLI	122.81	**0.009**	129.18	0.766
MetS (number parameter)	51.39	**<0.001**	80.10	0.376

BMI, Body Mass Index; WC, Waist Circumference; SBP, Systolic Blood Pressure; DBP, Diastolic Blood Pressure; HDL, High-Density Lipoprotein; LDL, Low-Density Lipoprotein; VAI, Visceral Adiposity Index; FLI, Fatty Liver Index; MetS, Metabolic Syndrome. A p value in bold type denotes a significant difference (p < 0.05).

To compare the relative predictive power of the cardio-metabolic indices and MetS, three multiple linear regression analysis models with oncological parameters (tumor grading, metastasis, and disease status) were performed and reported in **Table 7**. Model 1 compared the relative predictive power of grading G1/G2 on cardio-metabolic indices and MetS. In this model MetS entered at the first step (*p* < 0.001), followed by FLI (*p* < 0.001); VAI was excluded. Model 2 compared the relative predictive power of metastasis on cardio-metabolic indices and MetS. In this model, MetS entered at the first step (*p* < 0.001), followed by FLI (*p* < 0.001); VAI was excluded. In model 3, the disease status was better predicted by VAI (*p* = 0.014); MetS and FLI were excluded (**Table 7**).

**Table 7:** Multiple regression analysis models (stepwise method) with tumor aggressiveness and cardiometabolic indices and MetS.

Parameters	Multiple Regression analysis			
***Model 1—Tumor Grading-***	**R^2^**	***β***	***t***	***p* value**
MetS	0.257	0.514	6.19	**<0.001**
FLI	0.138	0.381	4.27	**<0.001**
*Variable excluded: VAI*				
***Model 2—Metastasis-***	**R^2^**	***β***	***t***	***p* value**
MetS	0.120	0.358	3.97	**<0.001**
FLI	0.100	0.330	3.61	**<0.001**
*Variable excluded: VAI*				
***Model 3—Disease Status-***	**R^2^**	***β***	***t***	***p* value**
VAI	0.031	0.336	2.49	**0.014**
*Variable excluded: MetS and FLI*				

VAI, Visceral Adiposity Index; FLI, Fatty Liver Index; MetS, Metabolic Syndrome. A p value in bold type denotes a significant difference (p < 0.05).

**Figure 5:**
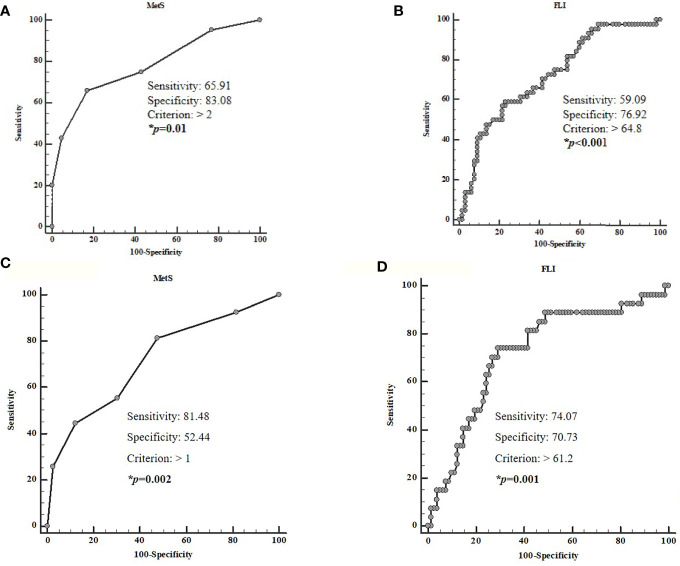
ROC analysis to determine the cut-off values of the MetS and FLI predictive of high grading **(A, B)** and the presence of metastasis **(C, D)**. A MetS >2 (*p* < 0.001, sensitivity 65.9%, specificity 83.1%, AUC 0.78, standard error 0.046; **A**) and a FLI > 64.8 (*p* < 0.001, sensitivity 59.1%, specificity 76.9%, AUC 0.72, standard error 0.050; **B**) could serve as thresholds for significant increased risk of G2 tumor. A MetS > 1 (*p* < 0.001, sensitivity 81.5%, specificity 52.4%, AUC 0.72, standard error 0.059; **C**) and a FLI> 61.2 (*p* = 0.001, sensitivity 74.1%, specificity 70.3%, AUC 0.72, standard error 0.058; **D**), could serve as a threshold for significantly increased risk of presence of metastasis. MetS, Metabolic Syndrome; FLI, Fatty Liver Index.

Four ROC analyses were performed to determine the cut-off values of the MetS and FLI predictive of high grading (G2) and presence of metastasis, respectively. A MetS> 2 (*p* < 0.001, sensitivity 65.9%, specificity 83.1%, AUC 0.78, standard error 0.046; **Figure 5A**) and a FLI >64.8 (*p* < 0.001, sensitivity 59.1%, specificity 76.9%, AUC 0.72, standard error 0.050; **Figure 5B**), could serve as thresholds for significant increased risk of G2 tumor. A MetS >1 (*p* < 0.001, sensitivity 81.5%, specificity 52.4%, AUC 0.72, standard error 0.059; **Figure 5C**) and a FLI >61.2 (*p* = 0.001, sensitivity 74.1%, specificity 70.3%, AUC 0.72, standard error 0.058; **Figure 5D**) could serve as a threshold for significantly increased risk of presence of metastasis.

## Discussion

In this cross-sectional, case–control, observational study, we evaluated the associations of VAI and FLI, as cardiometabolic indices, and MetS with tumor clinicopathological characteristics in a selected group of GEP-NET patients. The main result of the study is the positive association between the cardiometabolic indices and MetS with the clinicopathological characteristics of NET, independently of age and BMI. In addition, we have provided the cut-off values for the FLI and MetS to predict high grading of GEP-NET and the presence of metastasis.

Given the rarity and heterogeneity of GEP-NET, clinical trials designed to investigate the role of metabolic risk factors for these tumors are lacking. To the best of our knowledge, to date, this is the first study reporting differences in cardiometabolic indices in a selected group of GEP-NET patients compared to healthy controls matched for age, gender, and BMI.

The current prevalence of GEP-NET is 6.4 cases/100,000 inhabitants, with an increased incidence over the last four decades (2, 63). This increase was initially attributed to the improvement of diagnostic skills with the widespread use of advanced imaging techniques. However, the role of metabolic mechanisms underlying the etiology of GEP-NET has not yet been investigated before. Still, the potential contributions of different environmental factors, including metabolic dysfunctions, were mostly neglected as most evidence focused primarily on the genetics or molecular pathways of NET (64–66). Epidemiological data suggest that beyond the genetic influences, also environmental factors are involved in the increased incidence in GEP-NET (67). Indeed, only few retrospective evidence has addressed the potential association between MetS and GEP-NET (65, 68, 69), and these few studies were predominantly limited to pancreatic neuroendocrine tumors only (70, 71). In a recent case–control study, however, single risk factors of MetS, including visceral adiposity, high triglyceride levels, or hyperglycemia, were more present in GEP-NET patients compared to the control group (34).

NAFLD and MetS are well-established risk factors for different tumors; nevertheless, if these metabolic conditions are also risk factors for GEP-NET or if these conditions are able to negatively influence the clinicopathological characteristics of NET and consequently, disease behavior is yet to be fully established.

To the best of our knowledge, this is the first observation of GEP-NET patients with the highest values of VAI and FLI, and the presence of MetS are more likely to have higher-grade tumors or present advanced-stage disease at diagnosis with metastasis. In addition, VAI, FLI, and MetS were significantly associated with the three clinicopathological characteristics of GEP-NET included in this study.

These findings suggest that accurate metabolic profiling should be an integral part of the clinical evaluation of patients with GEP-NET and support a role for adiposity dysfunction and NAFLD, evaluated by VAI and FLI, respectively, and the presence of MetS as relevant risk determinants in GEP-NET patients. Similar associations were also shown for other types of tumors, such as esophageal cancer (72), colon and rectal cancer (73), thyroid cancer (74, 75), and prostate cancer (76).

However, this study has some limitations and some strengths that must be considered. Among the limitations, the cross-sectional nature of this study did not allow identification of any causal association between cardio-metabolic indices or MetS and GEP-NET characteristics and to clearly determine their prognostic value to predict GEP-NET clinical severity. Furthermore, the suggested cut-off value of FLI and MetS to identifying tumor aggressiveness should be viewed with caution until data in larger populations become available to perform an appropriate cross-validation. Moreover, we recognize how the liver biopsy is the gold-standard technique for identifying NAFLD. Hepatic biopsy is an invasive procedure burdened with rare but potentially life-threatening complications. However, FLI, although it is a surrogate marker of NAFLD, has largely proved to represent an easy and reliable screening tool to identify NAFLD (77, 78). The lack of a liver biopsy may prompt us to further investigate the association between cardiometabolic indices and MetS in GEP-NET patients.

However, the strengths of this study are several. First, the sample size was sufficiently large. In fact, we have calculated the sample size using 95% power, and the number of participants required was 102 (51 cases and 51 controls), while we used 218 (109 GEP-NET patients and 109 controls) individuals *i.e.* more than double those required. Second, the homogeneity of our sample population further strengthens the power of the study. In fact, in order to improve the power of this study, we increased the homogeneity of the cohort of NET patients by including only patients who were biochemically free of disease for more than 6 months without medical treatment, or treatment-naïve patients with non-functioning GEP-NET. In addition, all GEP-NET patients had a diagnosis of well-differentiated G1/G2 and were matched for age, sex, and BMI with a well-characterized control group.

## Conclusions

In conclusion, our findings report that the worsening of clinicopathological characteristics in GEP-NET is associated with visceral adiposity dysfunction, evaluated by VAI, NAFLD, evaluated by FLI, and the presence of MetS. These novel results, although requiring confirmation in larger scale clinical trials, help to fulfil an unmet clinical need and provide a breakthrough toward understanding the putative mechanisms leading to GEP-NET progression and increased prevalence. Finally, to address the clinical evaluation of cardiometabolic indices in GEP-NET patients might be of crucial relevance to establish targeted preventive and treatment interventions of NET-related metabolic comorbidities.

## Data Availability Statement

The raw data supporting the conclusions of this article will be made available by the authors, without undue reservation.

## Ethics Statement

The “Federico II” Medical School Ethical Committee has approved this cross-sectional case–control observational study (n. 201/17), which was conducted in accordance with the Code of Ethics of the World Medical Association (Declaration of Helsinki) for experiments involving humans. The patients/participants provided their written informed consent to participate in this study.

## Author Contributions

Conceptualization, LB, SS, and GM. Data curation, GM, GP, RMo, and RMi. Formal analysis, LB, GM, and SS. Funding acquisition, LB, AF, AC, and GM. Investigation, LB, GM, BA, RMo, RMi, and GP. Methodology, LB and GM. Project administration, LB and GM. Resources, LB. Software, LB. Supervision, LB, GM, AC, SS. Validation, AF, AC, SS, RMo, RMi, and LB. Visualization, LB. Writing—original draft, LB and SS. Writing—review & editing, LB, GM, AC, and SS. All authors contributed to the article and approved the submitted version.

## Conflict of Interest

The authors declare that the research was conducted in the absence of any commercial or financial relationships that could be construed as a potential conflict of interest.

## Abbreviations

NEN, Neuroendocrine neoplasms; NET, Neuroendocrine tumors; GEP, gastro-entero-pancreatic; WHO, World Health Organization; MetS, metabolic syndrome; NAFLD, non-alcoholic fatty liver disease; FLI, fatty liver index; BMI, body mass index; WC, waist circumference; TGs, triglycerides; GGT, glutamyltransferase; CVDs, cardiovascular diseases; VAI, Visceral Adiposity Index; HDL, high-density lipoprotein cholesterol; SDs, standard deviations; NEC, neuroendocrine carcinoma; SBP, Systolic Blood Pressure; DBP, Diastolic Blood Pressure; LDL, Low-Density Lipoprotein; MEN1, multiple endocrine neoplasia type 1; OR, Proportional Odds Ratio; IC, Interval Confidence; AIC, Akaike Information Criterion; ROC, Receiver operator characteristic; AUC, area under the curve.
